# Music-Based Interventions in Childhood Hearing Loss: A Comprehensive Narrative Review

**DOI:** 10.3390/children13040574

**Published:** 2026-04-21

**Authors:** Mirko Aldè, Letizia Casella, Umberto Ambrosetti, Stefania Barozzi, Eleonora Gandolfo, Federica Di Berardino, Diego Zanetti

**Affiliations:** 1 Department of Clinical Sciences and Community Health, Department of Excellence 2023–2027, University of Milan, 20122 Milan, Italy; umberto.ambrosetti@unimi.it (U.A.); stefania.barozzi@unimi.it (S.B.); eleonora.gandolfo@unimi.it (E.G.); federica.diberardino@unimi.it (F.D.B.); diego.zanetti@unimi.it (D.Z.); 2 Audiology Unit, Department of Specialist Surgical Sciences, Fondazione IRCCS Ca’ Granda Ospedale Maggiore Policlinico, 20122 Milan, Italy; letizia.casella@policlinico.mi.it

**Keywords:** musical auditory perception, musical training, music therapy, cochlear implants, hearing aids, hearing loss

## Abstract

**Highlights:**

**What are the main findings?**

• Children with hearing loss show reduced pitch and timbre perception.
• Musical training can improve auditory, linguistic, and cognitive skills.
• Music therapy studies are few, but available evidence suggests benefits that extend beyond auditory perception to emotional, social, and behavioral domains

**What are the implications of the main findings?**

• Music-based interventions may support broader communicative development by leveraging rhythmic strengths and enhancing auditory processing.
• Expanding and standardizing music therapy approaches could improve holistic care for children using cochlear implants or hearing aids.

**Abstract:**

**Background/Objectives:** Childhood hearing loss (HL) affects auditory, linguistic, and social development. Alongside conventional rehabilitation, music-based interventions have gained increasing attention for their potential to support both auditory and non-auditory domains. This narrative review aims to summarize current evidence on the use of music-based interventions in children with HL. **Methods:** A narrative review of the literature was conducted, examining studies involving pediatric cochlear implant or hearing aid users. Publications were categorized into three main areas: musical auditory perception, musical training, and music therapy. **Results:** Studies on musical auditory perception demonstrate persistent limitations in pitch and timbre perception in children with HL, while rhythmic abilities appear relatively preserved. Musical training interventions, particularly those targeting rhythm, have been associated with improvements in auditory perception, linguistic processing, and selected cognitive skills, although parental involvement and long-term designs remain limited. Existing literature on music therapy is scarce but suggests potential benefits extending beyond auditory skills to emotional regulation, social interaction, and quality of life. **Conclusions:** Music-based interventions represent a promising complementary approach in pediatric hearing rehabilitation. While musical training is more widely studied, music therapy is still underrepresented despite its holistic focus. Further structured studies are needed to define standardized protocols and outcome measures for music therapy in children with HL.

## 1. Introduction

Childhood hearing loss (HL) can markedly affect a child’s communication abilities, emotional growth, and social participation [1]. Limited auditory input often results in delays in speech development, difficulties in language comprehension, and reduced engagement with peers. These challenges may extend beyond communication, influencing academic performance, self-esteem, and overall quality of life [2]. Early, well-structured strategies remain essential to address the developmental vulnerabilities associated with pediatric HL. Music therapy has increasingly been recognized as a supportive intervention that addresses both auditory and non-auditory developmental domains [3]. Through structured musical experiences such as rhythmic activities, movement, singing, and instrumental interaction, music therapy stimulates multiple sensory pathways and fosters meaningful engagement [4]. Even in cases of moderate-to-profound HL, music can be perceived through vibrations, visual information, and tactile cues, allowing children to actively participate and express themselves creatively [5]. In addition to enhancing auditory awareness, music therapy supports emotional regulation, social interaction, and cognitive development. Musical activities promote attention, imitation, and turn-taking, which are skills closely associated with language acquisition [6]. Moreover, the enjoyable and motivating qualities of music may reduce anxiety and strengthen therapeutic relationships [7].

The present work provides a narrative review of the literature on the use of music-based interventions in pediatric patients with HL, including both cochlear implant (CI) and hearing aid (HA) users. The aim of this review is to offer an overview of the clinical interventions and practical experiences described to date on this topic. A comprehensive search was performed in the electronic databases PubMed/MEDLINE, Scopus, and Web of Science, including all publications available up to February 2026. The search strategy combined Medical Subject Headings (MeSH) and free-text terms related to HL and music-based interventions, using combinations of the following keywords: “hearing loss”, “cochlear implant”, “hearing aid”, “children”, “pediatric”, “auditory perception”, “music”, “musical training”, and “music therapy”. Boolean operators (AND/OR) were applied to refine the search and ensure broad coverage of relevant studies. Studies were considered eligible if they involved pediatric populations younger than 14 years with HL using CI and/or HA and investigated music-based interventions—musical auditory perception, musical training, or music therapy—provided that they reported original data. Research conducted exclusively in adult populations, articles not focused on music-related interventions, and non-original contributions such as editorials, commentaries, or conference abstracts lacking sufficient methodological detail were excluded. Specifically, the selection process was conducted in three stages. First, titles and abstracts identified through the database search were screened to exclude clearly irrelevant articles. Subsequently, the full texts of potentially eligible studies were retrieved and assessed for inclusion based on the predefined criteria. Finally, the reference lists of the included articles were manually screened to identify additional relevant studies. Given the narrative nature of this review and the heterogeneity of the included studies, no formal quality assessment or risk-of-bias tool was systematically applied; however, key methodological aspects, such as sample size, study design, and the presence of control groups, were taken into account during data interpretation. Overall, this work provides a narrative overview of the available literature rather than a formal systematic review, as the currently published studies are relatively few and differ substantially in terms of study design, interventions, and outcome measures. To facilitate a coherent structure, the analysis is organized into three main sections: musical auditory perception, musical training, and music therapy.

It is important to clarify the distinction between musical training and music therapy, as these terms are often used interchangeably despite referring to conceptually different approaches. Although they differ in several key aspects, the two modalities also share certain objectives and methodological elements. According to the World Federation of Music Therapy, music therapy is defined as “*the professional use of music and its elements as an intervention in medical, educational and everyday environments with individuals, groups, families or communities who seek to optimize their quality of life and improve their physical, social, communicative, emotional, intellectual and spiritual health and wellbeing. Research, practice, education and clinical training in music therapy are based on professional standards according to cultural, social, and political contexts*” [8]. From this definition, two key elements emerge that distinguish music therapy from other interventions involving sound and music: the presence of a specifically trained professional and the broad range of objectives related to the individual’s overall wellbeing, encompassing cognitive, emotional, behavioral, communicative, and interpersonal domains [9]. Interventions defined as musical training, by contrast, may be delivered by personnel without specific therapeutic training, sometimes by family members themselves or by teachers, and pursue objectives more narrowly related to the auditory domain, such as improvements in sound perception and in the discrimination of sounds and speech [10]. The use of programs or applications offering musical exercises and games falls predominantly within musical training; however, these tools may occasionally also be used in music therapy as a complement to other activities. Conversely, experiences defined as musical training often include playful auditory–musical activities that are also widely used in music therapy, such as singing, music listening, sound production with musical instruments, and exercises related to the perception of musical parameters and musical emotion [11]. As a matter of fact, the boundary between musical training and music therapy remains partially fluid in practice, as both approaches may employ similar techniques and, in some cases, pursue overlapping functional outcomes. This convergence reflects the shared use of music as a multisensory and relational medium, while differing primarily in therapeutic intent and professional framework. Accordingly, in the present review, an operational distinction was adopted based on the leading aim of the intervention and the clinical context in which it was delivered, in order to ensure conceptual clarity while acknowledging the existence of a continuum between these modalities. The key concepts and definitions of the terms used throughout this review are presented in Table 1.

## 2. Musical Auditory Perception in Children with Hearing Loss

In the articles addressing musical auditory perception, the primary focus is the evaluation of how children with HL perceive music and its constituent parameters. In the literature, musical auditory perception is described as “*the individuals’ abilities to process, analyze, remember, and predict acoustic patterns, auditory scenic content, and interval relations of syntax and semantics conveyed by music*” [12]. Music represents a primary perceptual stimulus for the human auditory system and is closely related to the early development of auditory and linguistic functions. The brain areas involved in musical and linguistic perception are anatomically adjacent and partially overlapping, suggesting a functional interaction between the two domains [13]. It has been observed that good musical perception is associated with better cognitive and emotional abilities. For this reason, recent research has aimed to improve the musical experience of CI users, while also promoting social integration and an improved quality of life [14]. However, the literature highlights significant limitations in the perception of musical elements in individuals with HL and in CI users. In particular, the temporal components of the acoustic signal (e.g., rhythm) are better encoded and therefore more easily perceived than the spectral and frequency components (such as timbre and pitch), which require a more accurate representation of the sound signal [13]. This occurs due to the reduced precision of spectral transmission of the acoustic signal, which is intrinsic to the electrical stimulation model of CI [15]. The technological limitations of CI result in significant difficulties in musical perception, mainly due to reduced spectral resolution and a limited dynamic range compared with normal hearing [16]. This limitation of CI often affects language development, as the perception of auditory information relies on variations in pitch, amplitude, and duration of speech [17]. Nevertheless, targeted musical training after cochlear implantation can enhance musical perception [18]. Since musical perception is shaped not only by acoustic and technological factors but also by emotional responses to musical stimuli [16,18,19], several neurobiological and developmental mechanisms have been proposed to explain why music-based activities may support auditory, linguistic, cognitive, and socio-emotional development in children with HL [20,21,22,23,24,25,26,27,28,29,30,31,32,33,34]. These mechanisms are summarized in Table 2.

### 2.1. Study Samples

Across most of the reviewed studies, sample sizes typically ranged from 27 to 75 children [13,14,15,17,19]. Two investigations deviated from this range: one study involved a notably small cohort of only five participants [16], while another included a substantially larger sample of 360 children [18]. Regarding methodological design, most studies included control groups that were exposed to the same experimental activities. These control groups were comparable to the HL groups in terms of both participant number and age range [14,16,17,18]. Only one study adopted a within-group comparison design, examining differences among children with HL without the inclusion of a separate control group [15]. In this case, participants were stratified according to age (older than 6 years versus 6 years or younger) and length of CI use (more than 18 months versus 18 months or less). In two investigations, an additional comparison group was included alongside the HL and normal-hearing cohorts. One study incorporated a group of children with autism spectrum disorder [19], while the other examined a group of children using bimodal auditory stimulation [13].

### 2.2. Intervention and Assessment Procedures

Studies examining musical parameters primarily assessed abilities such as singing performance, song and melody recognition, timbre discrimination, and responsiveness to rhythmic patterns [18]. In other investigations, participants were presented with pairs of sequential piano tones and were required to judge the pitch relationship between the notes, identifying whether the second tone was the same, higher, or lower than the first [15]. Several studies focused on emotional processing through music. These involved listening tasks using prerecorded violin excerpts [19] or selected musical stimuli specifically designed to evoke emotional responses [13]. Participants were then asked to associate the perceived emotions either with corresponding verbal descriptors [19] or with images depicting facial expressions representing those emotions [13]. In relation to speech perception, one study required children with HL to produce reading samples, which were subsequently compared with those of normal-hearing peers. The recordings were evaluated by trained listeners who were blinded to the participants’ hearing status to ensure objective assessment [17]. With regard to quality of life and neurophysiological measures, one investigation explored electroencephalographic (EEG) activity while participants viewed a music-based cartoon, providing insight into neural responses to musical stimuli [16]. Assessment approaches varied considerably across studies and included a wide range of standardized tools and measurement techniques. These comprised the Profiling Elements of Prosody in Speech-Communication (PEPS-C), which evaluates receptive and expressive prosodic skills; Diagnostic Analysis of NonVerbal Accuracy 2 (DANVA-2), a measure of nonverbal emotion recognition; and the Contour and Interval subtests of the Montreal Battery of Evaluation of Amusia (MBEA), which assess melodic contour processing and interval discrimination [17]. Additional tools included the Perception of Emotions and Movement in Music test (PEMM), designed to probe emotional and movement-related responses to musical stimuli; the Peabody Picture Vocabulary Test (PPVT) and Expressive Vocabulary Test (EVT), which measure receptive and expressive vocabulary; the Autism Diagnostic Observation Schedule (ADOS), a standardized assessment of social-communication behaviors; and the Clinical Evaluation of Language Fundamentals (CELF), a comprehensive measure of language abilities [19]. Auditory and music-related outcomes were captured using the Category of Auditory Performance (CAP), a functional hearing scale; the Speech Intelligibility Rating (SIR), which evaluates the clarity of speech production; the Musicality Rating Scale (MRS), a clinician-reported measure of musical behaviors; and the Subjective Assessment of Music Enjoyment (SAME), which captures listener-reported enjoyment and engagement with music [14]. Additional methodologies included EEG rhythm monitoring [16] and the “Musical Ears Evaluation Form for Professionals” [18], a structured tool for documenting musical perceptual abilities in clinical or educational settings.

### 2.3. Summary of Evidence

The evidence emerging from these studies consistently supports the effectiveness of music in therapeutic approaches for children with CI, as greater exposure to music and longer duration of CI use are associated with better musical auditory perception [14,15]. Furthermore, improvements in musical perception appear to play a meaningful role in alleviating language-related difficulties in children with HL, reflecting underlying neuroplastic adaptations associated with CI use [12]. Such changes highlight the potential of music-based approaches to support broader communicative development. Despite these positive effects, children with CI generally demonstrate lower performance in the perception of pitch, timbre, and melody, as well as in the recognition of musical emotions, particularly those relying on spectral sound information [14,19]. In addition, evidence suggests that music may be perceived as less enjoyable by children with CI compared to their normal-hearing peers [16], and that these children may experience greater difficulties with prosodic aspects of speech [17]. Importantly, enhancing musical abilities in children with HL has been linked to improvements in overall quality of life. Gains in musical skills may be accompanied by advances in auditory–verbal abilities and pitch discrimination, consistent with principles of neural plasticity. These findings underscore the importance of promoting musical engagement in children with CI through the identification and implementation of effective musical interventions and targeted auditory perception training [18].

## 3. Musical Training in Children with Hearing Loss

Among the different intervention approaches designed to enhance auditory perception or to compensate for hearing-related limitations, a considerable body of research highlights the use of musical training as an effective means of supporting perceptual, linguistic, and cognitive development in children using CI or HA [15,35,36,37,38,39,40,41,42,43,44,45,46,47]. Musical training refers to guided engagement in musical activities, typically delivered in educational or recreational contexts rather than as formal therapy. It utilizes auditory and musical stimuli with the aim of strengthening auditory perception, increasing familiarity with hearing devices, and supporting the use of residual hearing capacities [10]. Across the selected studies, considerable variability was observed in participant age ranges, group structures, levels of parental involvement, and the settings in which training was delivered. These variations largely stem from methodological heterogeneity and differing program objectives.

### 3.1. Study Samples

The studies on pediatric musical training generally involved relatively small samples, most often ranging between 10 and 30 participants. The majority of participants were of school age, although the age distribution varied considerably across studies and was not always clearly reported. In several investigations, samples included approximately 23–29 participants [15,35,36,37,38], with children typically aged between 5 and 11 years; only a few studies also included younger participants aged 1–4 years [35,39]. Other studies enrolled smaller cohorts of 7–18 participants [40,41,42,43,44,45,46,47], and some incorporated control groups to allow comparison with typically hearing (TH) peers or alternative intervention conditions [41,44,46,47]. Overall, the available evidence primarily concerns school-aged children, while research involving younger participants remains limited [40].

In a small number of studies, family members were actively involved in administering exercises at home, highlighting the potential role of the family environment in supporting musical training activities [41,43]. With regard to study design, two main types of control were identified. The first is “between-group” control, used in two studies, in which participants on a waiting list received no intervention during the initial experimental phase. This approach allows for a longitudinal comparison of training effects and, in some cases, also serves as a normative reference [35,41]. The second type is “within-group” control, in which the same participants are exposed over time to different interventions, such as an artistic activity or various musical training tasks, enabling internal comparisons within the group [37,38,44,47].

### 3.2. Intervention and Assessment Procedures

The intervention modalities were varied, but certain groupings can be identified according to specific criteria. Several studies focused on selected parameters of musical perception, aiming to enhance auditory–musical competencies [15,40,41,43,44]. Specifically, these interventions addressed the recognition, discrimination, and reproduction of rhythms and pitches. Within this category, one study investigated how previous musical education could positively influence the recovery of these abilities [15]. In another study, the intervention was based on musical production through singing, measuring parameters such as pitch and intonation [40]. Other studies focused on linguistic and phonological support through musical training [39,42,45]. In these cases, music was primarily used as a supportive tool for linguistic exercises rather than as an explicitly rehabilitative intervention. Accordingly, the outcomes reported across studies reflect their primary objectives and may not capture the full range of potential effects. In one project, music therapy combined with musical training primarily aimed at improving the child’s psychosocial and relational development [47]. Finally, one study used musical elements as a stimulus to enhance executive functions [37].

### 3.3. Summary of Evidence

Several studies investigated the effects of musical training targeting auditory perception [15,35,36,38,39,41,46]. Results indicate that training is associated with observable improvements in timbre and pitch perception, and that the duration of musical education is positively correlated with pitch discrimination performance. Effects on speech abilities were not consistently observed, while in home-based programs administered by family members, participants showed improvements in note discrimination, with more pronounced gains in musical skills than in linguistic abilities [41,43]. Studies focusing on rhythm demonstrated positive effects on linguistic abilities, such as improved adaptation and responsiveness in verbal interaction, greater phonological accuracy, and increased sensitivity to temporal regularity. In particular, when phonological production was accompanied by regular rhythmic training, improvements in syntactic processing were observed [42,44,45]. Subjective reports suggested that, beyond gains in language and perceptual abilities, participants also experienced psychosocial benefits, particularly in emotional regulation, interpersonal relationships, and broader aspects of quality of life [46,47]. Studies analyzing musical abilities in the absence of a specific intervention revealed that children with CI exhibit significant difficulties in vocal production with accurate intonation, especially in pitch variation, while rhythmic skills remain relatively preserved [40].

## 4. Music Therapy in Children with Hearing Loss: Approaches and Evidence

A total of five publications describing music therapy approaches for pediatric CI or HA users were identified [46,48,49,50,51]. Three studies described music therapy techniques applicable to this population [48,49,50]. One study reported a two-part survey assessing healthcare professionals’ knowledge of music therapy for children with HL and parents’ perceptions of its effectiveness following participation in a music therapy program [51]. The remaining study presented a clinical investigation in which music therapy was delivered as part of multimodal measures [46]. Although the intervention also included structured musical training and app-based home exercises, it is discussed within the music therapy domain due to the central role of a certified music therapist, the group-based interactive format, and the primary focus on psychosocial and quality-of-life outcomes. A summary of the included studies on musical training and music therapy is provided in Table 3.

### 4.1. Music Therapy Approaches

Barton and Robbins began their work by outlining the potential of musical experiences to enhance developmental abilities in normally hearing children and subsequently described methods and music therapy approaches tailored to this population, with the aim of fostering auditory learning in early childhood [50].

Given the potential of music to positively influence brain regions involved in non-musical functions, such as attention, emotion, cognition, behavior, communication, and perception [52,53], two therapeutic frameworks for initiating auditory learning have been proposed: the Comprehensive Music Therapy Approach [54] and the Hybrid Approach to Pediatric Language Intervention [55]. This was followed by a presentation of specific musical and linguistic intervention techniques in which music was used as a supportive tool for working with children with CI.

Bruscia identified two main strategies that clinicians could employ within the Comprehensive Music Therapy Approach [54]: an “outcome-oriented strategy”, in which the therapist defines specific objectives based on the patient’s needs and applies evidence-based practices to address them, and an “experience-oriented strategy”, in which the therapist allows the patient to guide the process, using musical experiences as a means of revealing and shaping therapeutic goals. Specifically, four principal therapeutic methods are described [54]. The “re-creative method” involves the child singing or playing pre-existing songs, thereby supporting the development of specific skills and behaviors. The “improvisational method” allows the child to create melodies and rhythms spontaneously, offering an important channel for emotional expression, particularly in individuals with limited verbal abilities. The “receptive method” is based on active listening, with the child responding verbally or through movement, and can be used to stimulate imagination or to target specific auditory functions, such as associating movement with particular sounds or instruments. Finally, the “compositional method” involves the co-creation of musical material with the therapist, sometimes resulting in recordings or audiovisual products, and is particularly useful for expressing and documenting thoughts, feelings, and experiences.

Fey suggested that “natural” language interventions aligned with real-life communicative experiences increased the likelihood that skills would generalize to other contexts, supporting their long-term maintenance. Naturalness may be compromised in high-intensity, repetitive, exercise-based practices. Fey therefore proposed a “hybrid” approach to pediatric language intervention, balancing structured exercises aimed at acquiring basic musical skills with natural interactions in which the child actively “makes music,” similar to the Comprehensive Music Therapy Approach [55].

Barton and Robbins suggested a range of techniques in which music is used to support work with children with CI, organizing the interventions according to age group [50]. For infants and toddlers, they emphasize the use of infant-directed singing and songs integrated throughout the child’s daily routine, along with teaching nursery rhymes or songs associated with gestures, and highlighting the importance of moments of silence. For preschool children, the approach includes daily singing, the use of simple and accessible musical instruments, and the introduction of sprechgesang, a vocal technique that blends speech and singing. It also involves singing familiar melodies using different voices, incorporating musical games to reinforce listening skills, and using music to strengthen relational actions and conceptual understanding. With primary school children, the techniques shift toward transforming directive phrases into melodies or rhythmic patterns, working on synchronization with a steady beat, and using music as a tool to reinforce rhythm recognition. For upper primary school children, music is used more abstractly and expressively, such as conveying non-musical information through musical means, creating experiential opportunities for social interaction, encouraging musical composition to express feelings or personal experiences, and training skills like rhythm and frequency discrimination.

In the study by Gfeller and colleagues [48], possible music therapy interventions for preschool CI users were described, aimed at supporting listening skills, speech production, language development, socialization, cognition, emotional, behavioral, and motor development. Similar to the approaches outlined by Barton and Robbins [50], the authors emphasized adapting early-childhood musical activities to the specific needs of CI users and selecting “CI-friendly” musical stimuli. After describing empirical findings from studies on musical perception in CI users (covering rhythm, timbre, pitch, melody, song lyrics, singing, enjoyment, and musical participation), Gfeller and colleagues suggested collaboration between the music therapist and the broader clinical team [48]. This ensured that the therapist fully understood the child’s specific functioning while also providing assessments in previously unexamined areas, such as social skills. Consistent with Bruscia’s framework [54], key therapeutic targets included listening, speech production, and language development, addressed through the structured use of instruments, singing, and movement. The music therapist worked on key speech sounds by selecting specific instruments and encouraging the child to pronounce their names. Vocabulary and linguistic concepts were reinforced by using instruments to represent shapes, sizes, and physical characteristics, providing concrete examples. Interactive communication was also trained by asking the child questions about the instruments [48].

Movement-based activities were described as essential not only for motor development but also for supporting communication goals, echoing principles of naturalistic intervention proposed by Fey [55]. These activities could be directed toward sound detection, discrimination, identification, auditory comprehension, or language development. Musical stimuli effectively guided the speed and mode of movement, while the child’s movements could promote attention, perception, and understanding of a given stimulus. Because movement activities primarily supported listening skills, therapists were required to pay particular attention to providing clear verbal instructions at an appropriate pace, demonstrating new activities simply and clearly, or offering verbal instructions without background music. Lip-reading was also effective when the therapist’s face was visible. Singing, on the other hand, required coordination of the same vocal mechanisms used in spoken communication. Singing in group settings demanded attentive listening, which could be particularly challenging for children with HL. Songs were selected to reinforce vocabulary, speech sounds, and concepts relevant to the child’s daily life and individualized educational or therapeutic plan. Collaboration with audiologists or speech-language pathologists was recommended for setting objectives and selecting song texts, which could be adapted to meet specific needs [48].

Esmaeilzadeh and colleagues described the Auditory–Verbal Music Play Therapy (AVMPT), an educational approach based on listening and aimed at cognitive development [49]. This method integrated principles from Auditory–Verbal Therapy (AVT), play therapy, and music therapy. As originally articulated by Pollack in 1970 [56], AVT applies techniques, strategies, and procedures to promote spoken language development through listening, supporting children (even very young ones) in hearing, understanding, and communicating verbally. Parents are involved as primary facilitators of listening development through active and consistent participation in the child’s individual AVT sessions. According to the Association for Play Therapy (APT), play therapy is a structured, theory-based therapeutic approach in which trained clinicians use the inherent therapeutic value of play to help individuals address psychosocial difficulties and support healthy development. Esmaeilzadeh et al. reported that this type of intervention could benefit areas such as problem solving, behavior, mood, language, social skills, learning, and the expression of feelings and fears. It was considered particularly suitable for children with HL, as it enabled them to express thoughts and emotions through the language of play in a context relying more on the visual channel than on spoken language [49]. In AVMPT, core principles of AVT, play therapy, and music therapy are integrated. Specifically, the music therapist teaches linguistic elements through musical games. Target words are introduced using strategies such as sing-song voice, modeling correct language, repetition, proximity to the microphone, repeating the word in various phrases, intoning or rhythmically producing it, and pairing verbal production with games or activities. Through these methods, the child may more effectively learn the target word and subsequently apply it in everyday communicative situations.

### 4.2. Summary of Evidence

Comincini and Del Piccolo conducted a two-part survey [51]. The first part involved administering a questionnaire to 60 professionals who treat children with HL participating in music therapy (including audiologists, speech therapists, otolaryngologists, general practitioners, and psychotherapists), aimed at understanding their opinions regarding this type of approach. The second part involved a group of 8 parents of children aged 3 to 6 years with varying degrees of HL who had attended weekly music therapy sessions for one year. Among professionals, 42% had not treated children with HL involved in music therapy and therefore did not express an opinion. Of the remaining participants, only a minority had direct clinical experience, but overall perceptions were positive. The main reported benefit was improved psychological well-being, whereas effects on sound discrimination, language, and prosody were considered less relevant. No resistance toward music therapy was observed among professionals.

In the parent survey, information was collected on participation characteristics (duration, modality, parental involvement) and perceived outcomes. Children were generally willing to participate in sessions, and parents reported predominantly positive emotional states following therapy, including happiness, calmness, and increased engagement. Overall, music therapy was perceived as effective in supporting auditory–behavioral rehabilitation and enhancing interaction with the environment.

Lo and colleagues investigated the benefits of a 12-week musical training program for children with HL, with a particular focus on psychosocial outcomes and quality of life [46]. Two groups of participants were tested: a group of children with sensorineural HL (SNHL) and a control group. The SNHL group undergoing the program consisted of 14 children (7 females and 7 males), aged between 6 and 9 years, with bilateral prelingual moderate-to-profound SNHL. Among them, 8 were bilateral CI users, 4 bimodal users, and 2 bilateral HA users. Eleven children started the musical training, while 3 completed only the baseline measurements. Of the 11 who began the training, only 9 completed all test sessions. The control group included 16 TH children (7 females and 9 males), aged 6–9 years. The program included weekly 40 min group music therapy sessions, with groups of 4–5 children led by a registered music therapist from the Speech and Hearing Clinic at Macquarie University. The approach followed was Nordoff-Robbins Creative Music Therapy, emphasizing interactive, social, and group-based activities in a non-verbal context. Additionally, 3 home sessions per week were prescribed, lasting 15–30 min, with online musical activities delivered via the MusicFirst Junior app, adaptable by the music therapist to meet specific objectives.

Assessments were conducted at baseline, after the 12-week training, and at a 12-week follow-up post-intervention. Results showed that, at baseline, children with HL had more emotional and behavioral difficulties and fewer prosocial behaviors than their TH peers. After the intervention, internalizing problems significantly decreased, and this improvement was maintained at follow-up. Total difficulties also improved after the program, although this effect was not sustained over time. Importantly, after training, children with HL reached levels comparable to their TH peers in these areas. No significant changes were observed in externalizing problems or prosocial behavior. In terms of quality of life, children with HL initially scored slightly lower than their peers, and no significant improvements were observed following the intervention. Similarly, self-reported hearing-related quality of life remained lower than that of TH children, with no significant changes over time. However, parent-reported outcomes indicated that there were notable improvements in overall quality of life, particularly in emotional well-being, learning, confidence, and the ability to accomplish tasks, while no meaningful changes were found in physical health or vitality.

## 5. Limitations

This narrative review has several limitations that should be acknowledged. First, the available literature on music-based interventions in children with HL remains heterogeneous in terms of study design, sample size, participant characteristics, and outcome measures. Moreover, the number of available studies is still relatively limited, particularly for music therapy interventions in pediatric populations. Given this heterogeneity, a formal systematic synthesis or quantitative comparison of results would not have been methodologically appropriate. This variability limits direct comparison across studies and precludes firm conclusions regarding efficacy, particularly for music therapy interventions, which are still underrepresented in the literature. In contrast, evidence from adult populations is comparatively robust and well established [57,58,59,60,61,62,63,64,65]. Second, many of the reviewed studies involve small samples, short program periods, and a lack of long-term follow-up, which limits the ability to assess the durability and broader applicability of the observed effects and reduces the statistical power of the findings. Although control groups are frequently included, methodological aspects such as blinding procedures and effect size reporting are inconsistently described. Consequently, causal inferences regarding the impact of musical exposure remain limited. In addition, parental involvement, home practice adherence, and baseline musical exposure are often insufficiently controlled or reported, representing potential confounding factors. Third, outcome measures vary widely and frequently rely on parent- or self-reported questionnaires, which may be subject to reporting bias. Objective auditory or neurophysiological measures are less commonly employed, particularly in studies focusing on psychosocial outcomes. A further limitation is that audiological management (e.g., HA or CI fitting, optimization, and usage) was inconsistently reported across studies, making it difficult to determine whether the observed benefits of music-based interventions are influenced by appropriately fitted amplification or occur independently of it. The lack of studies comparing music-based family interventions with other family-centered approaches also limits the ability to disentangle the specific effects of music from those related to increased parent–child interaction and enriched listening environments. Finally, the inclusion of hybrid measures combining music therapy and musical training may have contributed to some overlap between categories, reflecting the current lack of standardized definitions in this field. These limitations highlight the need for future well-powered, longitudinal, and controlled studies using standardized outcome measures to better define the role of music-based interventions, and particularly music therapy, within pediatric hearing rehabilitation.

## 6. Clinical Perspectives: Development of an Early Music Therapy Program

Building on the evidence synthesized in this review, it is also important to consider how music-based approaches can be translated into structured clinical practice. Against this background, a pilot music therapy initiative was recently introduced at our pediatric audiology outpatient clinic within the Fondazione IRCCS Ca’ Granda Ospedale Maggiore Policlinico in Milan (Italy). The project involves an early group music therapy intervention addressed to preschool-aged patients with CI or HA, starting from one year of age. Groups consist of a minimum of 3 to a maximum of 5 children, each accompanied by a parent who actively participates in the session. Sessions are held on a weekly basis, last 40 min, and are conceived within a medium- to long-term perspective (minimum duration of therapy: one year). The intervention is performed by a qualified professional music therapist, supported by a music therapist in training, and alternates structured activities of auditory–musical stimulation with moments of free instrumental and vocal improvisation. The approach is playful and multisensory, allowing ample space for expressiveness and interpersonal interaction. During the sessions, an observer external to the therapeutic setting (either a medical resident or a music therapist in training) is present and observes through a one-way mirror. Treatment monitoring is currently based on two instruments developed by the outpatient clinic team: an evaluation grid completed at the end of each session by the therapist together with the external observer, and a questionnaire periodically completed by parents and, separately, by the therapist. These tools aim to explore different domains, including engagement, relational and emotional aspects, as well as auditory, expressive, and communicative skills. Parental questionnaires are also intended to explore perceived changes across several developmental areas, including motor, linguistic, cognitive, relational, emotional, sound-musical, and expressive-creative domains.

This clinical initiative is currently being implemented as part of the broader rehabilitative pathway offered to children with HL using CI or HA. As the program expands, systematic longitudinal monitoring and structured outcome analyses will be essential to delineate its specific contributions to auditory, communicative, emotional, and relational development. In a broader perspective, initiatives of this kind may help shape future evidence-informed frameworks for integrating music therapy into multidisciplinary pediatric hearing rehabilitation, ultimately contributing to the definition of standardized, developmentally sensitive clinical protocols.

## 7. Conclusions

Drawing on studies concerning musical auditory perception in children with HL, it is evident that music plays an important role as a primary perceptual stimulus, closely linked to the early development of auditory and language functions, as well as to cognitive and emotional growth. Limitations in musical perception among children with HL, particularly CI users, are well documented and primarily affect timbre and pitch perception, whereas rhythmic abilities appear relatively preserved. However, engagement in activities aimed at enhancing musicality has been reported to improve musical perception, leading to greater enjoyment of music and, consequently, enhanced overall quality of life. Music-based interventions have also been associated with improved auditory perception and verbal skills. Therefore, identifying adequate and effective musical intervention strategies that engage pediatric patients early and positively, and that allow for medium- to long-term treatment durations, is crucial. In this context, musical training interventions are far more prevalent than music therapy interventions. Notably, studies on musical training predominantly involve school-aged children, while research on preschoolers is very limited. Consequently, parental involvement is minimal, reported in only one study, where parents’ role was limited to supervising online musical exercises at home. Within the musical training domain, the presence of control groups is significantly lower than in studies on musical auditory perception. In some cases, treatment efficacy is assessed via pre- and post-intervention tests administered to the children or their parents. Identifying control groups with homogeneous characteristics (e.g., age or clinical history) is often challenging, which may explain the frequent use of within-subject control designs. From a musical perspective, key outcomes include improvements in timbre and pitch perception (the latter positively correlated with the duration of musical education) and note discrimination abilities. Positive effects on linguistic and syntactic skills appear more evident in training programs focused on rhythm.

Regarding music therapy, the literature is sparse, with a limited number of studies available. Among these, only one reports a clinical trial, which employed an integrated approach combining group music therapy and musical training. This was a short-term intervention, partially compatible with music therapy principles, which typically require medium- to long-term application. Consequently, the efficacy of music therapy in pediatric populations with HL remains insufficiently documented. This may reflect limited awareness among healthcare professionals of the potential benefits of music therapy in children—not only for auditory perception but also for attention, emotion, cognition, behavior, and communication. Such benefits appear closely linked to the naturalness and enjoyment of the activity, which integrates auditory perception with other perceptual channels and provides ample scope for movement, play, and interpersonal interaction. Although limited in number, parent perceptions of children undergoing music therapy are qualitatively positive, encompassing auditory, behavioral, and social aspects. The sporadic availability of studies on music therapy effectiveness highlights the need for increased experimental music therapy projects within healthcare facilities serving children with HL, involving team-based planning and outcome assessment. Finally, careful consideration of evaluation methods is warranted, given the wide variety of pre-existing and ad hoc scales and questionnaires used in the reviewed studies. Notably, assessments in musical training interventions focus primarily on auditory perception, musical parameters, and language, whereas those in music therapy also consider broader aspects of quality of life. The use of standardized assessment scales, while useful for objective and widely comparable evaluation, should be complemented by questionnaires and rating grids capturing all dimensions of music therapy interventions, including socialization, behavior, emotional regulation, and overall patient well-being.

## Figures and Tables

**Table 1:** Key concepts and definitions related to music and hearing loss.

Term	Definition	Functional Role
Cochlear implant	A surgically implanted device that bypasses damaged structures of the cochlea and delivers patterned electrical stimulation directly to the auditory nerve.	To restore access to sound and enable speech and music perception within the constraints of current technology.
Hearing aid	A wearable device that amplifies and shapes the acoustic signal for individuals with HL.	To enhance audibility, supporting the perception of environmental sounds, speech, and music.
Musical auditory perception	The ability to process, analyze, remember, and predict the acoustic patterns conveyed by music, including temporal elements such as rhythm and spectral features such as pitch and timbre.	To support auditory, cognitive, and linguistic development by engaging attention, memory, and sensorimotor integration.
Music-based interventions	Structured use of musical activities within therapeutic, educational, or rehabilitative frameworks.	To modulate auditory processing, linguistic development, cognitive functions, emotional regulation, and social participation.
Music therapy	A clinical discipline in which trained and credentialed music therapists use structured musical experiences.	To achieve individualized developmental, communicative, emotional, behavioral, and psychosocial health outcomes.
Musical training	Systematic, goal-directed instruction in musical activities, typically without a therapeutic mandate and often delivered by non-clinical personnel.	To strengthen musical and auditory competencies, such as auditory discrimination, fine motor skills, and multisensory integration.
Pediatric hearing rehabilitation	A set of coordinated clinical and educational interventions designed for children with HL, involving auditory devices, therapeutic strategies, and communication support approaches.	To improve language, learning, and social participation through optimized auditory access and communication support.

HL = Hearing loss.

**Table 2:** Potential neurobiological and developmental benefits of music-based interventions in children with hearing loss.

Domain	Potential Mechanisms	Proposed Benefits
Attention and executive functions	Engagement of fronto-temporal and fronto-striatal networks during musical interaction.	Improved attentional control, working memory, and cognitive flexibility.
Auditory cortical plasticity	Repeated exposure to structured sound patterns enhances neural responsiveness within auditory cortical networks.	Improved sound discrimination, pitch perception, and auditory pattern recognition.
Cross-modal sensory integration	Interaction between auditory, motor, and somatosensory neural systems during musical activities.	Improved multisensory integration and support for speech–motor coordination.
Emotional and social processing	Activation of limbic and reward-related neural systems during musical interaction.	Enhanced emotional expression, social engagement, and parent–child interaction.
Language and phonological processing	Overlap between neural circuits for music and speech processing.	Support for phonological awareness and early language development.
Motivation and engagement in rehabilitation	Music-driven reward and dopaminergic modulation of learning circuits.	Increased participation in and adherence to rehabilitative activities.
Temporal processing and rhythmic entrainment	Synchronization of neural oscillations with rhythmic stimuli.	Enhanced speech perception, prosody recognition, and timing of auditory processing.

**Table 3:** Characteristics and key findings of studies on musical training and music therapy in children with hearing loss.

Study (Author, Year)	Population	Intervention Type	Study Design	Key Outcomes
Chen et al., 2010 [15]	Prelingually deaf children with CI	Musical training	Controlled experimental study (between-group)	Improved pitch perception correlated with training duration.
Koşaner et al., 2012 [35]	Preschool children with CI	Musical training	Prospective interventional study (pre-post)	Improved auditory–musical skills.
Welch et al., 2015 [36]	Children with CI/HA	Musical training	Pilot interventional study (single-group)	Improved pitch perception and singing skills.
Mason et al., 2021 [37]	Deaf children (mostly CI users)	Musical training	Interventional study (within-subject design)	Improved executive functions.
Aksu et al., 2024 [38]	Preschool children with CI	Musical training	Prospective interventional study (pre-post)	Improved phonological awareness.
Dastgheib et al., 2013 [39]	Children with CI/HA	Musical training	Interventional study (pre-post)	Improved speech and language outcomes.
Xu et al., 2009 [40]	Prelingually deaf children with CI	Musical training	Controlled cross-sectional study	Significant deficits in vocal pitch accuracy; rhythmic production is comparable to normal-hearing controls.
Yucel et al., 2009 [41]	Children with CI	Musical training	Longitudinal interventional study (between-group control)	Improved musical perception (note discrimination).
Cason et al., 2015 [42]	Prelingually deaf children with CI	Musical training	Experimental study (within-subject design)	Rhythmic priming improved phonological accuracy (especially in CI users).
Rocca, 2015 [43]	Children with HL (pre- and post-CI)	Musical training	Observational descriptive study	Improved early communicative and listening behaviors.
Hidalgo et al., 2017 [44]	Children with HL (predominantly CI users)	Musical training	Experimental study (within-subject design)	Improved speech timing and auditory–linguistic processing.
Bedoin et al., 2018 [45]	Children with CI	Musical training	Controlled experimental study	Improved syntactic processing.
Lo et al., 2020 [46]	Children with CI/HA	Music therapy (multimodal program led by a certified therapist, integrating musical training)	Prospective controlled longitudinal study	Improved speech-in-noise perception, timbre perception, and prosodic processing; associated psychosocial benefits.
Lo et al., 2022 [47]	Children with CI/HA	Musical training	Prospective controlled longitudinal study	Reduced internalizing difficulties; improved parent-reported quality of life.
Gfeller et al., 2011 [48]	Preschool children with CI	Music therapy	Clinical descriptive study	Reported benefits in listening, speech, language, social, and emotional domains.
Esmaeilzadeh et al., 2013 [49]	Children with CI/HA	Music therapy	Interventional study (pre-post)	Improved language, cognition, and social interaction.
Barton & Robbins, 2015 [50]	Children with CI	Music therapy	Theoretical/clinical framework	Describes developmentally structured therapeutic techniques.
Comincini & Del Piccolo, 2013 [51]	Children with CI/HA; professionals and parents	Music therapy	Survey-based observational study	Perceived improvements in psychological well-being and engagement.

CI = Cochlear implant; HA = Hearing aid; HL = Hearing loss.

## Data Availability

Not applicable.
